# Factors Affecting Depression and Its Relation to Sleep Quality among Parents of Type 1 Diabetes Patients

**DOI:** 10.3390/healthcare11070992

**Published:** 2023-03-30

**Authors:** Mi-Kyoung Cho, Mi Young Kim

**Affiliations:** 1 Department of Nursing Science, Chungbuk National University, 1 Chungdae-ro, Seowon-gu, Cheongju KR28644, Republic of Korea; 2 College of Nursing, Hanyang University, 222 Wangsimni-ro, Seongdong-gu, Seoul KR04763, Republic of Korea

**Keywords:** depression, sleep quality, resilience, type 1 DM, parents

## Abstract

This study investigated factors affecting depression (CES-D) among parents of patients with type-1 diabetes mellitus (T1DM), a chronic disease that requires constant management. A complex set of factors influence depression in parents and thus requires further research. This is a cross-sectional descriptive study. A survey on related variables was conducted on 217 parents of patients with T1DM. The collected data were analyzed using the PASW Statistics program, and factors influencing participants’ depression were identified through stepwise multiple regression. The results show that three variables exerted a significant effect on depression (source of information, resilience–personal competence, and Pittsburgh sleep quality index score), and all the variables explained a majority of the variance in depression. The results indicate that the parents of patients with T1DM were less depressed when the source of information was personal, when their resilience–personal competence was high, and when their Pittsburgh sleep quality index (PSQI) score was low. Interventions targeting parents of patients with T1DM should be performed with positive information on how to overcome diabetes in their children, increase resilience–personal competence, and increase sleep quality.

## 1. Introduction

Type 1 diabetes mellitus (T1DM) is an autoimmune condition that results from the destruction of beta cells in the pancreas [1]. T1DM is one of the most common chronic diseases in youth [2]. It requires constant adaptation and management, which is a complex and challenging process that includes diet control, exercise, insulin injection, and blood glucose level monitoring [3]. 

Due to the nature of T1DM, disease-related self-management should be performed daily. When children are young, parents are heavily involved in the disease’s management, which results in psychosocial distress for them and their children. Furthermore, despite the critical role of parents, who primarily serve as primary care providers in caring for T1DM children, parents are also vulnerable to being negatively affected by their children’s illnesses. It has been reported that approximately half of the parents of T1DM children experience negative psychosocial problems due to their children’s disease [4]. Specifically, parents of children with T1DM have been reported to develop distress, stress, anxiety, depression, and posttraumatic disorders [5]. These negative psychosocial experiences affect parents’ abilities to cope. A previous study has shown that the more depressed and anxious the parents are, the more they try to cope emotionally and use maladaptive coping strategies such as avoidance [4]. Parents’ negative emotions are not limited to themselves but also affect their children’s disease management; a previous study reported that the psychological distress of parents of chronically ill children affects their children’s health outcomes [6]. As negative emotions such as depression in parents of T1DM children can also affect their children’s disease management, paying attention to such negative emotions is needed. Therefore, this study aims to investigate the degree of depression, a representative psychological aspect, in the parents of T1DM children and the factors influencing it. 

Moreover, the issue of sleep quality in the parents of children with T1DM is often overlooked. Chronic sleep disturbance is widespread in parents of children with T1DM, and negatively affects daily function and well-being [7]. Sleep is essential to activate physical function and maintain health, and low-quality sleep can cause fatigue, impaired memory and concentration, restlessness, and negative emotions such as anxiety [8]. Furthermore, the effects of sleep are significant; sleep deprivation can increase the risk of cardiovascular disease, diabetes mellitus, obesity, cancer, hypertension, dyslipidemia, physical risks, impaired immune system function, and body coping ability, and affect mortality [9]. Thus, sleep is closely related to health, and it has been reported that parents of T1DM children have low sleep quality [10]. 

Resilience, a positive asset, is related to depression and sleep problems, to which parents of children with T1DM are vulnerable. As resilience is a positive adaptation to stressful situations and represents a mechanism for overcoming difficult experiences [11], it can be used as a positive asset to overcome negative factors. 

Therefore, this study aims to examine the effects of parents’ general characteristics, including resilience and children’s disease-related characteristics, on depression among parents caring for their children with T1DM. Parents are divided into good and poor sleepers to explore whether there are any differences in the factors influencing depression. 

## 2. Materials and Methods

This cross-sectional descriptive study aimed to verify the factors influencing depression in parents of children with T1DM.

### 2.1. Participants

The participants in this study were parents of patients with T1DM who were enrolled as members of the T1DM online community. The selection criteria were parents whose children had been diagnosed with T1DM for more than six months, who had read the research notice posted in the online community, and who voluntarily participated. Only one parent per household was required to answer the survey questionnaire. 

The number of participants required for this study was calculated using the sample size calculation program G Power 3.1.9.4 to determine the minimum sample size required to perform multiple linear regression [12] with the following parameters: seven characteristic variables of participants as independent variables (age, gender, religion, occupation, number of children, education on diabetes, source of information on diabetes), five characteristic variables of children with T1DM children (age, gender, duration of illness, HbA1c, complications), and two variables of resilience and global PSQI scores, as well as a two-sided significance level (α) of 0.05, statistical power (1-β) 0.95, and median effect size (f2) 0.15 [13]. The number of participants required with a 10% withdrawal rate was 214. The online survey with a consent form was shared with the T1DM online community, which was available only to approved and registered members. Efforts were made to avoid a potential selection bias during participant recruitment. A total of 217 participants participated in this study, and none withdrew.

### 2.2. Assessments

#### 2.2.1. Characteristics of the Participants

The characteristics of the target group were age, gender, religion, occupation, number of children, education on diabetes, and source of information on diabetes. Age, gender, duration of illness, complications, and recent HbA1C values were collected as characteristics of children with T1DM. Gender, a categorical variable, took the response “male” or “female”, and religion, occupation, education on diabetes, and complications were responded to with ”yes” or “no’’, and age corresponded to the year of birth. For the duration of the illness, the diagnosis year was written, and for HbA1C (%), the value was written in numbers. Personal source means information from the parent of the patient with type I DM. Expert source means information from the doctor or nurse based in the hospital. 

#### 2.2.2. Depression

Depression was measured using the self-reported simple screening test for depression (CES-D) developed by Radloff [14] to evaluate depressive symptoms. It was validated in Korea by Cho and Kim [15] and the items related to depression experienced over the past 7 days have a 4-point scale with a total of 20 items. All items were scored on a scale of 0 to 3 (0: extremely rare or less than 1 day, 1: sometimes or 1–2 days in a week, 2: often or 3–4 days in a week, 3: most often, more than 5 days in a week). The total score ranged from 0 to 60, and the higher the sum of the scores, the more severe the depression symptoms. Among these items, three positive items (No. 5, 10, and 15) were treated as inverse items.

For depression, the average CES-D score of Koreans was 15.6 points, which was higher than that of Americans at 9.1 points [16]. In a study by Cho and Kim [15], 21 points were used to select the CES-D score of Koreans, which is the standard suggested to set the sensitivity to 95% or more, the false-negative rate to less than 5%, and the primary target for depression to 25%. At the time of tool development, Cronbach’s α = 0.85 in Radloff’s study [14], 0.89~0.93 in the study by Cho and Kim [15], and 0.929 in this study.

#### 2.2.3. Global PSQI

The Pittsburgh Sleep Quality Index (PSQI), developed by Buysse et al. [17], has been used as an effective tool to measure sleep quality and disturbance. It was validated in Korea by Choi et al. [18]. This scale is a self-report questionnaire that measures the quality of sleep and degree of discomfort in sleep duration over the past month at the time of the test. The first four questions were in the form of the respondents directly entering the time they went to bed, the time it took them to fall asleep, the time they woke up, and the time they actually slept. In addition, various factors occurring during sleep, the frequency of taking sleeping pills, and the frequency of work interruption were scored on a scale of 0 to 3 (0: 0 times a week, 1: less than 1 time, 2: 2 times a week, 3: 3 times or more). It consisted of reporting the subjective sleep quality from “very good” to “very bad”. Each item was divided into seven components: subjective sleep quality, sleep latency, sleep time, daily sleep efficiency, sleep disturbance, use of sleeping pills, and daytime dysfunction. A score of 0 to 3 was assigned again. The global PSQI score is the overall PSQI component score, which ranges from 0 points (no sleep problems) to 21 points (severe sleep disturbance). The cutoff point of the global PSQI score was 5; if it was less than 5, the person was classified as a good sleeper, and if it was 5 or more, they were classified as a poor sleeper. The reliability of Buysse et al. [17] was 0.83 at the time of development, Cronbach’s α = 0.782 in the study by Choi et al. [18], and the reliability is 0.773 in this study.

#### 2.2.4. Resilience

In this study, the resilience scale developed by Wagnild and Young [19] was used as a tool and validated by researchers in the Korean context. Resilience is a psychological construct observed in some individuals that accounts for success despite adversity. Resilience reflects the ability to bounce back, to beat the odds, and is considered an asset in human characteristic terms [20]. Personal competence and acceptance of self and life are subscales of resilience. Resilience consisted of two sub-factors of personal competence (17 items) and acceptance of self and life (8 items), with a total of 25 items ranging from “disagree (1 point)” to “agree (7 points)”. Resilience was evaluated on a 7-point Likert scale of the total resilience score ranging from 25 to 175, with a score below 121 representing low resilience, 121 to 145 representing moderate resilience, and scores of 145 or higher representing moderately high to high resilience. In the study by Wagnild and Young [19], the reliability of Cronbach’s alpha at the time of development was 0.91, and it was 0.945 in this study.

### 2.3. Ethical Considerations

Participants were informed of the purpose and procedure of the study, the rights of the participants, and that anonymity was guaranteed. In addition, only individuals who read the online consent form that included a description of the study and voluntarily consented to participate were able to participate in the survey.

### 2.4. Data Collection

Data were collected using an online self-report questionnaire from August 19 to 31, 2020. The researchers explained the purpose and procedure of the study to the managers of the T1DM online community website and asked for their cooperation and consent. Information regarding the participant recruitment for the online survey was posted on the Notices section of the T1DM online community website by website managers. Research participants read the purpose and procedure of the study on the website notice section and clicked the online survey URL to answer the online consent form and then participated in the survey. Data were collected on T1DM parental characteristics, T1DM child characteristics, depression, sleep quality, and resilience.

### 2.5. Statistical Analysis

The collected data were analyzed using the PASW Statistics program (version 25.0; SPSS, IBM, New York, NY, USA). The characteristics of the participants were analyzed using frequency, percentage, mean and standard error, and depression, the global PSQI score, and resilience were analyzed with mean and standard error and minimum and maximum using descriptive statistics. Participants were divided into good sleepers and poor sleepers according to the global PSQI score, and the difference in depression according to participant characteristics was analyzed by an independent *t*-test. One-way ANOVA, Kruskal–Wallis test, and post hoc analysis were performed using the Scheffe test. Pearson’s correlation was used to examine the correlation between the global PSQI score, resilience, and depression in parents of children with T1DM. Factors influencing depression in individual parents with children with T1DM were identified through stepwise multiple regression. The cutoff for statistical significance in the present study was *p* < 0.05.

## 3. Results

### 3.1. Characteristics of the Participants

Of the total participants, the average age of children with T1DM was 12.53 (SD = 6.41) years, 116 (53.5%) were female, two children with T1DM was 124 (57.1%), the average duration of disease was 4.76 (SD = 4.64) years, HbA1c was 7.20% (SD = 1.48, and 9 (4.1%) children had experienced complications. Among the parents, 203 (93.5%) received education on diabetes, and 190 (87.6%) of the sources of information on diabetes were personal, such as club cafes and the Internet. Resilience was moderate in 95 (43.8%) participants. The characteristics of participants were similar in good sleepers and poor sleepers (Table 1).

### 3.2. Descriptive Statistics of Variables

The average score for depression of all parents was 11.61 (SE = 0.70, range: 0–48), for good sleepers, 5.47 (SE = 0.72, range: 0–26), and for poor sleepers, 13.80 (SE = 0.85, range: 0–48) points. As for the Global PSQI score, the average score of all participants was 8.00 (SE = 0.24, range: 1–18), for good sleepers, 4.04 (SE = 0.14, range: 1–5), and for poor sleepers, 9.41 (SE = 0.24, range: 6–18) points. For resilience, the average of all subjects was 128.59 ± 1.40 (range: 68–175), for good sleepers, 136.14 (SE = 2.26, range: 106–175), and for poor sleepers, 125.90 (SE= 1.66, range: 68–162), showing overall moderate resilience (Table 2). 

### 3.3. Differences in the Degree of Depression according to Characteristics of the Participants

According to the characteristics of the participants, participants without a job and education on diabetes had a higher degree of depression (t = 2.04, *p* = 0.043, t = 3.25, *p* = 0.001). Depression was higher when the number of children with T1DM was three or more than when there were one or two (F = 6.44, *p* = 0.040). Depression was higher when the children’s HbA1c level was more than 8.0% than when it was between 6.5% and 7.9% (F = 3.66, *p* = 0.027). Depression was higher in the low resilience than in the moderate and high resilience groups (F = 34.68, *p* < 0.001). Among poor sleepers, depression was significantly higher in participants who answered that they do not have a religion (t = 2.47, *p* = 0.015), participants who responded that they did not receive education on diabetes (t = 2.71, *p* = 0.008), when the source of information on diabetes was ‘experts’ (t = −2.47, *p* = 0.015), when participants had one or two children with diabetes compared to three or more (F = 7.45, *p* = 0.024), and those who had lower resilience compared to moderate and high resilience groups (F = 24.90, *p* < 0.001). There was no difference in the degree of depression according to the other participant characteristics and among the good sleepers (Table 3).

### 3.4. Correlation of Depression and Other Variables

For all participants, depression was positively correlated with global PSQI score (r = 0.61, *p* < 0.001) and negatively correlated with total resilience score (r =−0.47, *p* < 0.001), personal competence in resilience (r = −0.47, *p* < 0.001), and acceptance of self and life in resilience (r =−0.44, *p* < 0.001). Furthermore, the global PSQI and total resilience scores were negatively correlated (r = −0.38, *p* < 0.001). Poor sleepers had the same correlation results as all participants. Good sleepers showed a negative correlation only with depression and the resilience sub-factor acceptance of self and life (r =−0.27, *p* = 0.040, Table 4).

### 3.5. Factors Influencing Depression

To identify factors influencing depression in parents of children with T1DM, a stepwise multiple regression analysis was conducted using age, age of children, duration of disease, serum HbA1c, and resilience that can affect depression as continuous variables. The categorical variables of sex, sex of child, religion, job, number of children, complication, education on diabetes, and source of information on diabetes were used as dummy variables. There were no multicollinearity problems among the independent variables (Table 5).

The factors found to influence depression in parents of children with T1DM were the source of information on diabetes (B = −3.28, t = −2.06, *p* = 0.041) and personal competence resilience sub-factor (B = −0.20, t = −4.80, *p* < 0.001), with an explanatory power of 44.1% for depression variance (F = 57.32, *p* < 0.001). When the source of information on diabetes was personal, and the personal competence resilience sub-factor was high, parental depression decreased; the higher the Global PSQI score, the higher the degree of depression.

Among good sleepers, the participants’ characteristic variables, resilience, global PSQI score, duration of disease (B = −0.31, t = −2.19, *p* = 0.033), and acceptance of self and life resilience sub-factor (B = −0.32, t = −2.61, *p* = 0.012) were found to have a significant effect on depression. All the variables explained 12.6% of the variance in depression (F = 4.95, *p* = 0.011). Among the same group, parental depression decreased when the disease duration was long, and the acceptance of self and life resilience sub-factor was high.

Among poor sleepers, subject characteristics, resilience, and global PSQI score were entered into the model, and three variables, religion (B = −2.89, t = −2.18, *p* = 0.031), the personal competence resilience sub-factor (B = −0.23, t = −4.58, *p* < 0.001), and global PSQI score (B = 1.62, t = 6.87, *p* < 0.001), were found to have a significant influence on depression, and the model’s explanatory power was 40.4% (F = 36.64, *p* < 0.001). Parental depression decreased when religiousness and the personal competence resilience sub-factor were high, whereas parental depression increased when the global PSQI score was high.

## 4. Discussion

This study was conducted to examine the factors influencing depression in parents of children with T1DM and to determine whether there were differences in the factors influencing depression depending on good and poor sleep. The results of this study are as follows. In the depression model of parents of children with T1DM, the degree of depression was lower when the source of information on diabetes was personal rather than an expert. When the personal competence resilience sub-factor was high and when the overall sleep quality was high, the explanatory power of the model consisting of these variables was 44.1%. In this state of loss, it can be seen that managing negative emotions is vital for parents. In this regard, a study on parents of children with chronic diseases reported that parents with direct or indirect experiences of overcoming the same disease as their children had higher post-traumatic growth [21]. As such, a parent’s psychological distress is caused not only by the child’s T1DM diagnosis. Thus, emotional support is crucial, as indicated in a study by Bowes et al. [22]; they identified the importance of ongoing emotional support for parents. Furthermore, knowledge-oriented information from experts in hospitals is essential; however, emotional support in the form of personal exchanges and support from people who can be role models is also important. 

Moreover, this study shows that the higher the personal competence resilience sub-factor among the sub-domains of resilience, the lower the degree of depression. This finding supports a previous study [23] on the mediating role of resilience in the relationship between negative life events and depression. Resilience, as construed by Wagnild, comprises five essential characteristics: meaningful life (purpose), perseverance, self-reliance, equanimity, and existential aloneness (i.e., coming home to oneself) [24]. It is suggested that a lack of these protective factors influences the depression of parents caring for children with T1DM. The authors of [11] reported that resilience represents a variety of protective factors that are important for understanding health and disease, treatment, and healing processes and is a protective factor that makes individuals more capable of dealing with adverse events. In particular, among the resilience sub-factors, personal competence was found to be an influencing factor in this study. The resilience scale (RS) included a “personal competence” subscale and an “acceptance of self and life” subscale [19]. As Neill and Dias [25] defined it, personal competence involves such statements as “keeping interested in things is important” and “I have self-discipline”, beliefs that are thought to influence the practical management of diabetes. 

Furthermore, the results of this study show that the quality of sleep affects depression, a finding consistent with that of a study on the general population [26], which stated that depressive symptoms worsened when the quality of sleep was low. In particular, parents of children with T1DM may have more sleep disturbance factors than the general population with respect to disease management, such as nighttime blood glucose monitoring and hypoglycemia during sleep [27]. As sleep is essential for activating physical function and maintaining health, a lack of sleep can lead to fatigue, impaired memory and concentration, restlessness, and anxiety [8]. It is essential to assess whether parents of children with T1DM have sleep problems. In some cases, depression may make it difficult for them to sleep well, but it is believed that participants’ depression could be reduced by education or interventions that could help them sleep well, such as sleep education or sleep hygiene. 

This study examined the factors influencing depression in parents of children with T1DM by dividing them into good and poor sleep quality groups. The good sleeper group showed that the longer the duration of the disease and the higher the score in the resilience sub-factor acceptance of self and life, the lower the degree of depression, with the explanatory power of the model consisting of these variables at 12.6%. Given that, the longer the duration of the disease, the lower the degree of depression, and the shorter the duration of the disease, the higher the degree of depression. However, there are conflicting results regarding disease duration. The emotional aspect may vary depending on how parents spend the period in question rather than the absolute amount of time that has passed since diagnosis. Therefore, emotional support is also necessary for parents, considering that they may be vulnerable immediately after their children’s diagnosis. Continuous attention will be required thereafter, even if time has passed. The acceptance of self and life resilience sub-factor findings suggest that the emotional response may vary depending on how well the disease is accommodated. 

Meanwhile, in the poor sleeper group, if they are religious, their score on the resilience subfactor acceptance of self and life was high, overall sleep quality was high, and degree of depression was low, with the explanatory power of the model consisting of these variables being 40.4%. This finding reflects the importance of the spiritual aspect, which means it is also necessary to focus on this spiritual aspect when overcoming difficulties. Moreover, it can be seen that resilience plays a role in mitigating negative emotions, a finding in line with Smith et al. [28]. They reported that resilience plays a vital role in protecting and promoting mental health from risk factors such as stressful life events and enhances the ability to cope with crises by strengthening protective factors such as active coping. According to a study by Barnard et al. [7], which investigated the quality and quantity of sleep in parents of children with T1DM and the degree of impact on daily life, most parents found that waking up at night affected their daily functioning, which meant that they were more emotionally vulnerable if their sleep quality was low. Parents of children with T1DM manage their children’s disease but also have to socialize and sometimes work. Therefore, if sleep quality is low, it is impossible to spend the daytime efficiently because of fatigue, and it will be challenging to manage their children’s disease effectively. 

In addition to the factors examined so far, investigation of the differences in depression among parents caring for children with T1DM, according to general characteristics, reveals that depression was higher in parents who did not receive education on diabetes and that parents with one or two children are more likely to be depressed than parents with three children. Moreover, patients with glycated hemoglobin levels ≥ 8.0% show a higher degree of depression than those with lower glycated hemoglobin. This finding is consistent with that of a previous study [29], which found that parents’ quality of life was significantly lower when they did not receive education on diabetes, had only one child, or their child with the disease was the oldest. This finding suggests that education on diabetes is essential. Moreover, in light of the finding that a child’s sickness is expressed as losing a hoped-for typically healthy child [30], it can be suggested that when parents have one or two children, they feel a stronger sense of loss than when they have more than two children. Thus, it is necessary to consider the overall situation of parents’ families and to provide appropriate education. The results of this study will improve the understanding of depression, sleep, and resilience in parents of children with T1DM and provide fundamental data for developing interventions for depression.

The strength of this study is that it prepared basic data on depression and sleep of type 1 diabetic parents by conducting a study on type 1 diabetic parents, who are often overlooked. On the other hand, the limitation of this study is that it is a cross-sectional study and has limitations in clarifying cause and effect. Therefore, a cross-sectional study is suggested in the future. In addition, since it was not undertaken in a specific region, the study has the advantage that it can be relatively applicable nationwide. However, there may be a limit to external validity in terms of the fact that it was conducted in one country, Korea, and that only those who participated in the community could access the research participation. Therefore, caution is required in the interpretation of this study.

## 5. Conclusions

This study examined factors influencing depression among parents of children with T1DM. The results show that enhancing personal support and resilience and helping parents sleep well can lower depression among both parents. The results indicate that the positive internal power of resilience, maintenance of a good quality of sleep, and social support, including comfort and encouragement through emotional support or role models, are essential factors in reducing the negative emotions of parents of children with T1DM. These results are significant because they provide critical fundamental data for developing an intervention program to reduce depression and an integrated management program for parents. Furthermore, the psychosocial characteristics of the parents of T1DM children identified in this study can be used as evidence for self-management guidelines for T1DM in clinical practice.

## Figures and Tables

**Table 1:** Characteristics of the Participants (n = 217).

Characteristics		Total (n = 217)		Good Sleeper (n = 57)		Poor Sleeper (n = 160)	
		N (%)	Mean ± SD	N (%)	Mean ± SD	N (%)	Mean ± SD
Age (year)	<40	67 (30.9)	42.73 ± 5.87	13 (22.8)	44.40 ± 6.29	54 (33.7)	42.13 ± 5.61
	40~49	124 (57.1)		34 (59.7)		90 (56.3)	
	≥50	26 (12.0)		10 (17.5)		16 (10.0)	
Sex	Female	171 (78.8)		44 (77.2)		127 (79.4)	
	Male	46 (21.2)		13 (22.8)		33 (20.6)	
Religion	No	114 (52.5)		29 (50.9)		85 (53.1)	
	Yes	103 (47.5)		28 (49.1)		75 (46.9)	
Job	No	98 (45.2)		25 (43.9)		73 (45.6)	
	Yes	119 (54.8)		32 (56.1)		87 (54.4)	
Duration of disease * (year)	<5	131 (60.4)	4.76 ± 4.64	29 (50.9)	5.47 ± 4.98	102 (63.8)	4.50 ± 4.50
	5~10	64 (29.5)		20 (35.1)		44 (27.4)	
	>10	22 (10.1)		8 (14.0)		14 (8.8)	
Complication	No	208 (95.9)		52 (91.2)		156 (97.5)	
	Yes	9 (4.1)		5 (8.8)		4 (2.5)	
Education on diabetes	No	14 (6.5)		1 (1.8)		13 (8.1)	
	Yes	203 (93.5)		56 (98.2)		147 (91.9)	
Source of information on diabetes	Personal	190 (87.6)		49 (86.0)		141 (88.1)	
	Expert	27 (12.4)		8 (14.0)		19 (11.9)	
Age of child	≤12	119 (54.8)	12.53 ± 6.41	29 (50.9)	14.07 ± 6.39	90 (56.2)	11.98 ± 6.34
	13~18	73 (33.7)		17 (29.8)		56 (35.0)	
	≥19	25 (11.5)		11 (19.3)		14 (8.8)	
Sex of child	Female	116 (53.5)		32 (56.1)		84 (52.5)	
	Male	101 (46.5)		25 (43.9)		76 (47.5)	
Number of children	1	67 (30.9)		13 (22.8)		54 (33.7)	
	2	124 (57.1)		35 (61.4)		89 (55.6)	
	≥3	26 (12.0)		9 (15.8)		17 (10.7)	
HbA1c * (%)	<6.5	65 (30.0)	7.20 ± 1.48	17 (29.8)	6.98 ± 0.98	48 (30.0)	7.28 ± 1.61
	6.5~7.9	109 (50.2)		32 (56.1)		77 (48.1)	
	≥8.0	43 (19.8)		7 (12.3)		34 (21.3)	
Resilience	Low	72 (33.2)		11 (19.3)		61 (38.1)	
	Moderate	95 (43.8)		29 (50.9)		66 (41.3)	
	High	50 (23.0)		17 (29.8)		33 (20.6)	

* Missing value. Notes. SD: standardized deviation, Min: minimum, Max: maximum, HbA1c: Glycated hemoglobin.

**Table 2:** Descriptive Statistics of the Variables (n = 217).

Variables		Total (n = 217)				Good Sleeper (n = 57)		Poor Sleeper (n = 160)	
		Sum of Score		Converted Score		Sum of Score		Sum of Score	
		Mean ± SE	Min.~Max.	Mean ± SE	Range	Mean ± SE	Min.~Max.	Mean ± SE	Min.~Max.
CES-D		11.61 ± 0.70	0~48	0.58 ± 0.04	0~3	5.47 ± 0.72	0~26	13.80 ± 0.85	0~48
Global PSQI score		8.00 ± 0.24	1~18	1.14 ± 0.03	0~3	4.04 ± 0.14	1~5	9.41 ± 0.24	6~18
Resilience	Personal competence	87.94 ± 0.95	48~119	5.17 ± 0.06	1~7	93.49 ± 1.58	73~119	85.96 ± 1.12	48~113
	Acceptance of self and life	40.65 ± 0.49	20~56	5.08 ± 0.06	1~7	42.65 ± 0.76	30~56	39.94 ± 0.59	20~55
	Total score	128.59 ± 1.40	68~175	5.14 ± 0.06	1~7	136.14 ± 2.26	106~175	125.90 ± 1.66	68~162

Notes. SE: standardized error, Min: minimum, Max: maximum, CES-D: Center for Epidemiologic Studies Depression Scale, PSQI-C: Pittsburgh Sleep Quality Index.

**Table 3:** Depression According to Participant Characteristics (n = 217).

Characteristics		Total (n = 217)		Good Sleeper (n = 57)		Poor Sleeper (n = 160)	
		Mean ± SE	t or F or χ^2^ (*p*) (Scheffe)	Mean ± SE	t or F or χ^2^ (*p*) (Scheffe)	Mean ± SE	t or F or χ^2^ (*p*) (Scheffe)
Age (year)	<40	12.84 ± 1.35	0.79 (0.458)	6.92 ± 2.20	0.60 (0.553)	14.26 ± 1.53	0.08 (0.928)
	40~49	11.24 ± 0.90		5.03 ± 0.81		13.59 ± 1.11	
	≥50	10.23 ± 1.89		5.10 ± 1.12		13.44 ± 2.72	
Sex	Female	12.17 ± 1.31	1.71 (0.091)	5.68 ± 0.87	−0.53 (0.597)	14.42 ± 0.98	1.43 (0.155)
	Male	9.54 ± 0.81		4.77 ± 1.13		11.42 ± 1.66	
Religion	No	12.95 ± 1.03	2.04 (0.043)	4.76 ± 0.91	−1.02 (0.313)	15.74 ± 1.21	2.47 (0.015)
	Yes	10.14 ± 0.92		6.21 ± 1.11		11.60 ± 1.15	
Job	No	13.03 ± 1.06	1.85 (0.066)	6.60 ± 1.32	1.40 (0.166)	15.23 ± 1.25	1.55 (0.123)
	Yes	10.45 ± 0.92		4.59 ± 0.73		12.60 ± 1.15	
Duration of disease (year)	<5	11.89 ± 0.90	0.14 (0.870)	6.55 ± 1.19	1.63 (0.205)	13.41 ± 1.06	0.51 (0.604)
	5~10	11.06 ± 1.21		4.95 ± 0.96		13.84 ± 1.54	
	>10	11.55 ± 2.66		2.88 ± 0.83		16.50 ± 3.55	
Complications	No	11.74 ± 0.72	0.84 (0.400)	5.40 ± 0.77	−0.31 (0.756)	13.85 ± 0.86	0.34 (0.736)
	Yes	8.78 ± 2.57		6.20 ± 1.59		12.00 ± 5.40	
Education on diabetes	No	20.07 ± 2.88	3.25 (0.001)	3.00 ± 0.00	−0.46 (0.648)	21.38 ± 2.77	2.71 (0.008)
	Yes	11.03 ± 0.70		5.52 ± 0.73		13.13 ± 0.87	
Source of information on diabetes	Personal	11.18 ± 0.72	1.41 (0.169)	5.82 ± 0.80	−1.19 (0.239)	13.04 ± 0.88	2.47 (0.015)
	Expert	14.67 ± 2.37		3.38 ± 1.24		19.42 ± 2.65	
Age of child (year)	≤12	11.45 ± 0.91	1.14 (0.322)	6.48 ± 1.16	1.09 (0.343)	13.06 ± 1.10	0.67 (0.513)
	13~18	12.71 ± 1.32		4.71 ± 1.16		15.14 ± 1.54	
	≥19	9.16 ± 1.76		4.00 ± 1.04		13.21 ± 2.59	
Sex of child	Female	11.83 ± 1.00	0.33 (0.743)	4.81 ± 0.73	1.00 (0.329)	13.03 ± 1.17	−0.87 (0.388)
	Male	11.37 ± 0.98		6.32 ± 1.34		14.50 ± 1.23	
Number of children	1 ^a^	14.16 ± 1.36	6.44 (0.040) *	6.62 ± 1.99	1.23 (0.300)	15.98 ± 1.52	7.45 (0.024) *(a, b > c)
	2 ^b^	11.09 ± 0.92	(a, b > c)	4.60 ± 0.73		13.64 ± 1.15	
	≥3 ^c^	7.54 ± 1.15		7.22 ± 2.05		7.71 ± 1.43	
HbA1c (%)	<6.5 ^a^	10.78 ± 1.18	3.66 (0.027)	6.82 ± 1.70	2.45 (0.294) *	12.19 ± 1.44	2.25 (0.109)
	6.5~7.9 ^b^	10.54 ± 1.02	(c > a, b)	4.22 ± 0.73		13.17 ± 1.31	
	≥8.0 ^c^	15.39 ± 1.50		7.43 ± 2.21		17.03 ± 1.62	
Resilience	Low ^a^	17.75 ± 1.37	34.68 (<0.001) *	8.64 ± 2.04	2.50 (0.092)	19.39 ± 1.49	24.90 (<0.001) *
	Moderate ^b^	8.69 ± 0.79	(a > b, c)	4.55 ± 0.96		10.52 ± 0.98 (a > b, c)	
	High ^c^	8.32 ± 1.22		5.00 ± 1.02		10.03 ± 1.71	

Notes. * Kruskal–Wallis test, post hoc: Scheffe test, superscripts a, b, and c are groups for the Scheffe test. SE: standardized error, HbA1c: Glycated hemoglobin.

**Table 4:** Correlations between Variables (n = 217).

Variables	Sub-Factors	Total (n = 217)		Good Sleeper (n = 57)		Poor Sleeper (n = 160)	
		CES-D	Global PSQI Score	CES-D	Global PSQI Score	CES-D	Global PSQI Score
		r (*p*)					
Global PSQI score		0.61 (<0.001)		0.21 (0.121)		0.56 (<0.001)	
Resilience	Personal competence	−0.47 (<0.001)	−0.38 (<0.001)	−0.23 (0.080)	−0.18 (0.174)	−0.46 (<0.001)	−0.32 (<0.001)
	Acceptance of self and life	−0.44 (<0.001)	−0.34 (<0.001)	−0.27 (0.040)	−0.08 (0.564)	−0.43 (<0.001)	−0.35 (<0.001)
	Total score	−0.47 (<0.001)	−0.38 (<0.001)	−0.25 (0.056)	−0.15 (0.255)	−0.46 (<0.001)	−0.34 (<0.001)

Notes. CES-D: Center for Epidemiologic Studies Depression Scale, PSQI: Pittsburgh Sleep Quality Index.

**Table 5:** Factors Influencing Depression (n = 217).

Variables	Total (n = 217)			Good Sleeper (n = 57)			Poor Sleeper (n = 160)		
	B	SE	t (*p*)	B	SE	t (*p*)	B	SE	t (*p*)
Intercept	19.67	4.30	4.57 (<0.001)	20.57	5.39	3.82 (<0.001)	19.18	5.40	3.55 (0.001)
Religion (ref = No)							−2.89	1.33	−2.18 (0.031)
Duration of disease				−0.31	0.14	−2.19 (0.033)			
Source of information (ref = Expert)	−3.28	1.60	−2.06 (0.041)						
Resilience–Personal competence	−0.20	0.04	−4.80 (<0.001)				−0.23	0.05	−4.58 (<0.001)
Resilience–Acceptance of self and life				−0.32	0.12	−2.61 (0.012)			
PSQI	1.50	0.16	9.23 (<0.001)				1.62	0.24	6.87 (<0.001)
F (P)	57.32 (<0.001)			4.95 (0.011)			36.64 (<0.001)		
Adj. R^2^ (%)	44.1			12.6			40.4		
Tolerance	0.84~0.98			0.97			0.88~0.98		
VIF	1.02~1.19			1.03			1.02~1.13		
Durbin–Watson	1.93			1.80			1.96		

Notes. PSQI: Pittsburgh Sleep Quality Index, B: Unstandardized Regression Coefficient, SE: standard error, Adj. R^2^: adjusted coefficient of determination, VIF: Variance Inflation Factor.

## Data Availability

Not applicable.
